# Postbiotics from *Lacticaseibacillus rhamnosus* IOB820 Combat Obesity in HFD Mice by Modulating Gut Microbiota and Enhancing SCFA Production

**DOI:** 10.3390/nu17223525

**Published:** 2025-11-11

**Authors:** Xiaomin Feng, Hanlu Li, Jianxia Tian, Xuemei Han, Wu Liang, Feiliang Zhong, Xuegang Luo

**Affiliations:** 1 Key Laboratory of Industrial Fermentation Microbiology of the Ministry of Education, College of Biotechnology, Tianjin University of Science and Technology, Tianjin 300457, China; fxmxka@mail.tust.edu.cn (X.F.); lihanluha@163.com (H.L.); tian_tjx@mail.tust.edu.cn (J.T.); hanxuemei2024@163.com (X.H.); 2 Tianjin Key Laboratory of Edible Probiotics, Tianjin 300301, China; 13388059278@163.com

**Keywords:** *Lacticaseibacillus rhamnosus* IOB820, probiotics, obesity, gut microbiota, short-chain fatty acids

## Abstract

**Aims:** To evaluate whether *Lacticaseibacillus rhamnosus* (*L. rhamnosus*) IOB820 and its postbiotics can combat high-fat diet (HFD)-induced obesity, improve metabolic parameters, and modulate gut microbiota and systemic inflammation in a mouse model. **Methods:** Seventy 4-week-old male C57BL/6J mice were divided into a normal diet group, an HFD control group, two postbiotic dose groups, two live bacteria dose groups, and an orlistat control group. After 10 weeks of intervention with live *L. rhamnosus* IOB820 or its postbiotics, body weight, metabolic parameters (blood glucose, lipid profile, hepatic steatosis), pro- and anti-inflammatory cytokines (TNF-α, IL-6, IL-1β, IL-10), gut microbiota composition (α, β diversity and taxonomic shifts), and fecal short-chain fatty acid (SCFA) levels were assessed. **Results:** Both live *L. rhamnosus* IOB820 and its postbiotics significantly alleviated HFD-induced weight gain and improved metabolic outcomes. The treatments also reduced systemic inflammation, as indicated by decreased levels of TNF-α, IL-6, and IL-1β and elevated IL-10. These effects were accompanied by restoration of gut microbial diversity, enrichment of beneficial taxa, and increased fecal SCFA concentrations. **Conclusions:** *L. rhamnosus* IOB820 and its postbiotics effectively mitigate obesity and related metabolic disturbances in HFD-fed mice. Their beneficial effects are likely mediated through modulation of gut microbiota composition and enhancement of SCFA-driven anti-inflammatory responses.

## 1. Introduction

Obesity is a multifactorial chronic disease that contributes significantly to public health burdens globally, posing substantial challenges to both families and healthcare systems [1,2]. Epidemiological studies on obesity and its complications among Chinese adults reveal that 34.8% of adults are overweight, and 14.1% are obese. Studies further show that obesity prevalence is higher among men, individuals aged 35 to 39 years, and those from lower socioeconomic regions. The detection rate of fatty liver disease in obese individuals is as high as 81.8%, and as body mass index (BMI) increases, the risk of developing various complications rises significantly [3]. While genetic factors affect only a minority of individuals, the primary cause of obesity is an energy imbalance due to poor dietary habits and lifestyle choices, leading to abnormal or excessive fat accumulation [4,5]. Adipose tissue and the liver, as metabolically sensitive organs, play crucial roles in maintaining metabolic homeostasis [6,7,8]. Obesity is associated with chronic, low-grade inflammation in these tissues, where inflammatory cytokine signaling disrupts insulin pathways, impairing glucose uptake and promoting uncontrolled lipolysis. This process initiates a vicious cycle of ectopic lipid storage and insulin resistance [9,10]. Additionally, studies by Turnbaugh et al. have shown that diet-induced obesity correlates with an increased abundance of *Firmicutes* in the intestinal microbiota [11]. Intestinal bacteria facilitate bidirectional communication between the gut and adipose tissue, influencing energy metabolism, nutrient absorption, appetite regulation, and adipose tissue function [12,13]. Gut dysbiosis, often induced by obesity, can trigger systemic metabolic, inflammatory, and immune disorders, particularly in the context of impaired intestinal barrier function [14].

Currently approved drugs for obesity treatment, such as fenfluramine, temin, sibutramine, orlistat, and Qsymia, are associated with serious side effects and high relapse rates [15]. Moreover, Glucagon-Like Peptide-1 (GLP-1) receptor agonists effectively suppress appetite, enhance satiety, and delay gastric emptying, thereby promoting significant weight loss. Their lipid-lowering effects are primarily reflected in the reduction in triglycerides and improvements in the overall lipid profile. However, these treatments may also induce adverse gastrointestinal reactions [16]. Consequently, there is increasing interest in developing effective and safe weight loss therapies.

Probiotics are live microorganisms that, when administered in adequate amounts, confer health benefits to the host. Probiotic intervention serves as a crucial tool for improving host health [17]. Studies demonstrate that probiotics can promote weight loss and lipid reduction by modulating gut microbiota balance, influencing energy metabolism, and reducing fat accumulation [18,19]. Evidence suggests that probiotic supplementation significantly improves obesity-related metabolic markers. For instance, Kim et al. showed that *Lactiplantibacillus plantarum* APsulloc331261 exerts beneficial effects in high-fat diet (HFD)-induced obese mice, including reduced adiposity, improved glucose tolerance, ameliorated dyslipidemia, and enhanced hormone and adipokine levels [20]. Other research indicates that *Lacticaseibacillus rhamnosus* HF01 fermented yogurt ameliorates obesity-related phenotypes in mice, including weight reduction, improved blood lipid profile, and diminished hepatic lipid droplet formation [21]. Yang et al. observed that *Lacticaseibacillus rhamnosus* JL1 supplementation reduced body weight and lipid levels in HFD-fed mice, further supporting the potential of probiotics in treating metabolic disorders [22].

Postbiotics, metabolites or components derived from probiotics, offer advantages such as high stability, safety, and direct action. Balaguer et al. found that *Bifidobacterium lactis* subspecies BPL1 postbiotics exhibited fat-reducing properties in a Caenorhabditis elegans model [23]. Lim et al. demonstrated that *Lactiplantibacillus plantarum* K8 postbiotics prevent obesity and inflammation in HFD mice [24]. Additionally, growing evidence indicates that gut microbiota regulates diet and modulates host metabolism, producing metabolites that serve as microbial–host messengers [25,26]. As key metabolic regulators, SCFAs influence adipose tissue metabolism, lipid oxidation, β-cell function, and insulin secretion, suggesting a microbiota-SCFA-metabolism relationship and their potential as therapeutic targets for obesity and related diseases [27,28,29]. In these contexts, SCFAs inhibit histone deacetylases (HDACs) and activate receptors to maintain intestinal homeostasis. Similarly, gut microbiota ameliorates metabolism-related disorders by processing nutrients and regulating energy balance [30,31].

Although probiotics and postbiotics have shown promise in alleviating metabolic disorders, comparative studies on the efficacy of live bacteria versus their postbiotic derivatives in obesity management remain limited. Furthermore, the underlying mechanisms, particularly those involving gut microbiota remodeling and SCFA production, have not been fully elucidated. This study investigates the therapeutic potential of *Lacticaseibacillus rhamnosus* (*L. rhamnosus*) IOB820 and its postbiotics in preventing and treating obesity-related metabolic disorders by modulating gut microbiota and metabolite profiles in HFD mice. It aims to establish a theoretical foundation for novel probiotic applications in weight and lipid management, develop innovative therapeutic strategies for obesity, and provide microbial resources for functional food innovation.

## 2. Materials and Methods

### 2.1. Sample Pretreatment

Preparation of fermentation liquid: *L. rhamnosus* IOB820 was retrieved from the cryopreservation tube and inoculated into liquid MRS medium (Haibo Biotech, Qingdao, China) at a 3% inoculation rate, where it was cultivated to obtain the activated seed liquid. This activated seed liquid was then transferred into fresh liquid MRS medium at the same 3% inoculation rate to produce the fermentation seed liquid. The fermentation seed liquid was subsequently inoculated into liquid MRS medium at a 1% inoculation rate and cultured in a fermentation tank to produce the fermentation liquid.

Preparation of *L. rhamnosus* postbiotic powder: The *L. rhamnosus* fermentation broth from the fermenter was centrifuged at 12,000 rpm for 10 min and washed with physiological saline until no traces of MRS culture medium components remained. The bacteria were then inactivated by heating at 85–95 °C for 30–40 min. After inactivation, the broth was homogenized and spray-dried to obtain the IOB820 postbiotic powder. Spray drying conditions included a feed rate of 5–7 mL/min, inlet temperature of 170–200 °C, and outlet temperature of 50–70 °C.

### 2.2. Experimental Animals and Groups

Seventy specific pathogen-free (SPF) male C57BL/6J mice, aged four weeks, were housed in a controlled environment at 20–25 °C with a 12 h light/dark cycle (fluorescent light during the light phase). The animals were procured from the China National Medical Products Administration Academy (Beijing, China). Mice had ad libitum access to autoclaved water and standard rodent chow. Following a one-week acclimation period, mice were weighed and randomly assigned to seven experimental groups (*n* = 10 per group): Normal Diet control (NFD, 10% fat, 18% protein, 72% carbohydrate, Jiangsu Xietong Pharmaceutical, Nanjing, China), High-Fat Diet control (HFD, 60% fat, 20% protein, 20% carbohydrate, Jiangsu Xietong Pharmaceutical), Low-dose IOB820 postbiotics (L-POST), High-dose IOB820 postbiotics (H-POST), Low-dose live IOB820 (L-820, 1 × 10^8^ CFU/mL), High-dose live IOB820 (H-820, 2 × 10^9^ CFU/mL), and Orlistat positive control (OLYM, 10 mg/mL).

Each day at 9:30 AM, the mice were gavaged with 0.2 mL of the respective solution. The gavage volume was adjusted according to the body weight of the mice, and the bacterial solution was freshly prepared every day. The NFD and HFD groups received physiological saline (0.2 mL) with weekly volume adjustments based on individual body weight. This regimen was sustained for 10 consecutive weeks. Mice were euthanized using Isoflurane, and eyeball dissection was performed to collect whole blood into sterile centrifuge tubes. After 30 min of standing, the tubes were centrifuged at 3500 rpm for 15 min at 4 °C. The supernatant was collected as serum. Liver and epididymal adipose tissues were harvested. All animal procedures were conducted following the ARRIVE guidelines (https://www.nc3rs.org.uk/arrive-guidelines, accessed on 19 February 2024) and approved by the Academic Committee of Tianjin University of Science and Technology on 12 March 2024 (SWKL-20240312007). These guidelines strictly adhered to the ARRIVE principles.

### 2.3. Determination of Fasting Blood Glucose

Mice were gently placed on an iron grid, and approximately 1–2 mm of the tail tip was quickly cut off to collect blood for blood glucose testing. To minimize stress, the procedure was performed as gently as possible. Fasting blood glucose levels were immediately measured using clinically validated blood glucose test strips (Roche, Shanghai, China) and a calibrated glucometer, following the manufacturer’s protocol. All measurements were performed in duplicate within 2 min of blood collection to ensure analytical accuracy.

### 2.4. ELISA

Serum samples and mechanically ground liver tissue homogenates were analyzed for the quantitative determination of the following biomarkers: low-density lipoprotein cholesterol (LDL-C, A113-2-1, Nanjing Jiancheng, Nanjing, China), high-density lipoprotein cholesterol (HDL-C, A112-2-1, Nanjing Jiancheng), total cholesterol (TC, A111-1-1, Nanjing Jiancheng), triglycerides (TG, A110-1-1, Nanjing Jiancheng), tumor necrosis factor-α (TNF-α, ZC-39024, Shanghai ZCIBIO, Shanghai, China), interleukin-10 (IL-10, ZC-37962, Shanghai ZCIBIO), interleukin-6 (IL-6, ZC-37988, Shanghai ZCIBIO), interleukin-1β (IL-1β, ZC-37974, Shanghai ZCIBIO), aspartate aminotransferase (AST, ZC-38728, Shanghai ZCIBIO), and alkaline phosphatase (ALP, ZC-33964, Shanghai ZCIBIO). Analyses were performed using a multimode microplate reader with optimized wavelength settings for each assay. All procedures adhered strictly to the manufacturer’s kit instructions.

### 2.5. HE Staining

Mouse liver and epididymal adipose tissue specimens were fixed in 4% paraformaldehyde for 24 h at room temperature. Fixed tissues were subjected to sequential dehydration using graded ethanol (70%, 80%, 90%, 100%), followed by clearing with xylene for tissue transparency. Tissue blocks were embedded in paraffin, sectioned at a thickness of 4 μm using a rotary microtome, and dewaxed in xylene. Sections were rehydrated through descending ethanol concentrations (100%, 90%, 80%, 70%) and rinsed in distilled water. They were then stained with hematoxylin for 10 min, differentiated with acid ethanol, counterstained with eosin for 3 min, and dehydrated with ascending ethanol concentrations before clearing in xylene [32]. Sections were mounted with neutral balsam and examined under a bright-field microscope (Olympus, Tokyo, Japan).

### 2.6. RT-qPCR

RNA was extracted from mouse liver tissue using Trizol reagent and reverse transcribed into cDNA. RT-qPCR was performed using the SYBR Green system, and data were collected using the StepOne™ system. The method was employed for data analysis, with Glyceraldehyde-3-phosphate dehydrogenase (*GAPDH*) as an internal reference gene for relative quantification of Carnitine acyltransferase I (*CPT-1*) gene expression. Primer sequences are shown in Table 1. RT-qPCR was carried out with the following cycling conditions: 95 °C for 5 min, followed by 30 cycles of 95 °C for 30 s, 55 °C (*GAPDH*) or 57 °C (*CPT-1*) for 30 s, and 72 °C for 30 s, with a final extension at 72 °C for 10 min.

### 2.7. Determination of SCFAs

Cecal contents from mice were homogenized in sterile PBS (pH 7.4) at a 1:5 ratio (*w*/*v*), then centrifuged at 12,000× *g* for 15 min at 4 °C. The supernatant was collected, and SCFAs and other organic acids were extracted by acidifying the supernatant to pH 2–3 using 1 M HCl, followed by mixing with ethyl acetate (1:1 *v*/*v*) and vortexing for 2 min. After phase separation, the organic layer was collected, dried under a nitrogen stream, and re-dissolved in 50 μL methanol. Derivatization was performed using *N*-tert-butyldimethylsilyl-*N*-methyltrifluoroacetamide (MTBSTFA) at 70 °C for 30 min. Gas chromatography–mass spectrometry (GC-MS) was used for analysis [33]. SCFAs were quantified based on peak area ratios relative to calibration standards, which included acetic acid, propionic acid, butyric acid, isovaleric acid, valeric acid, glutamic acid, and succinic acid (Sigma-Aldrich, St. Louis, MO, USA).

### 2.8. 16s rDNA Sequencing

Genomic DNA was extracted from mouse colon contents using the CTAB/SDS method. Briefly, 200 mg of colon contents were mixed with 1 mL CTAB/SDS lysis buffer (containing 2% CTAB, 1% SDS, 100 mM Tris-HCl, pH 8.0, 20 mM EDTA, 1.4 M NaCl), ground in liquid nitrogen, and incubated at 65 °C for 1 h. After extraction with chloroform-isoamyl alcohol (24:1), DNA was precipitated with isopropanol and washed with 70% ethanol. DNA purity and concentration were assessed using a NanoDrop 2000 spectrophotometer, and integrity was verified via 1% agarose gel electrophoresis. The 16S rRNA V3-V4 region was PCR amplified using bacterial universal primers, and microbiome diversity was analyzed using Kruskal–Wallis and one-way analysis of variance (ANOVA) tests. Principal component analysis (PCoA) and linear discriminant analysis effect size (LEfSe) were used to analyze beta diversity and significant differences in microbial communities. Sequencing was performed using the Illumina NovaSeq 6000 platform.

### 2.9. Statistical Analysis

Data were analyzed using GraphPad Prism v9.0.0 software. Normality was assessed using the Shapiro–Wilk test. Results are presented as the mean ± standard deviation (SD). One-way ANOVA was used for comparisons between groups. Statistical significance was set as: ns (not significant, *p* > 0.05), * *p* < 0.05, ** *p* < 0.01, *** *p* < 0.001, **** *p* < 0.0001.

## 3. Results

### 3.1. L. rhamnosus IOB820 and Its Postbiotics Attenuate Weight Gain, Improve Fasting Blood Glucose, and Modulate Organ Indices in HFD Mice

**Figure 1:**
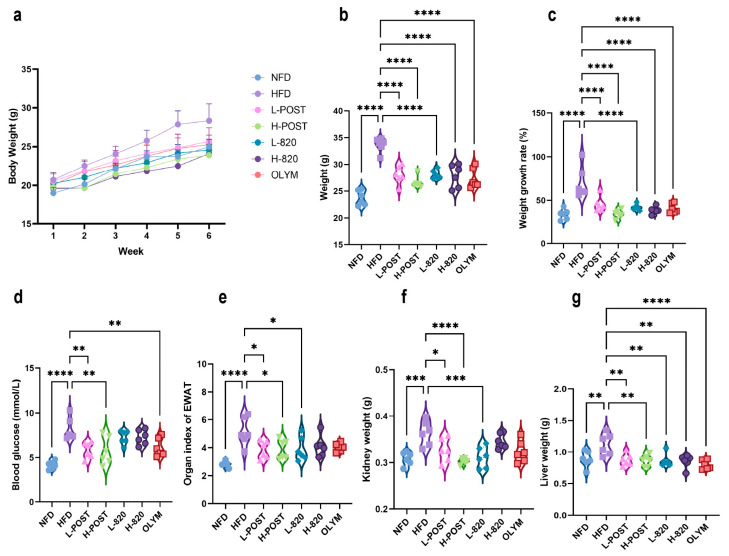
Effects of *L*. *rhamnosus* IOB820 and Postbiotics on Body Weight, Fasting Blood Glucose, and Organ Indices in HFD Mice. (**a**) Body weight progression during 10-week intervention; (**b**) Terminal body weight comparisons across experimental groups; (**c**) Percentage body weight gain relative to baseline; (**d**) Fasting blood glucose concentrations at endpoint; (**e**) Epididymal adipose indices (tissue weight/body weight × 100%) of epididymal adipose; (**f**) Kidney weight; (**g**) Liver weight. Statistical significance: * *p* < 0.05, ** *p* < 0.01, *** *p* < 0.001, **** *p* < 0.0001 vs. HFD group, *n* = 6.

Following 10 weeks of oral intervention, both *L. rhamnosus* IOB820 and its postbiotics exerted significant suppressive effects on weight gain in HFD mice. The H-POST group demonstrated superior efficacy, limiting weight increase to 38.9% ± 9.5%, a value comparable to that of the normal diet control group (NFD: 36.5% ± 5.7%) and significantly lower than the HFD group (67.5% ± 14.0%). Weight gains for other groups were as follows: LP (45.7% ± 6.7%), L820 (40.0% ± 4.2%), H820 (41.1% ± 6.5%), and OLYM (39.8% ± 9.4%) (Figure 1a–c). During the initial four-week treatment phase, no significant weight gain inhibition was observed in any intervention group. However, progressive suppression of weight gain became evident with prolonged treatment.

Post-intervention fasting blood glucose measurements revealed significantly higher levels in the HFD group (*p* < 0.0001 vs. NFD). Notably, both L-POST, H-POST, and OLYM achieved significant glucose-lowering effects (*p* < 0.01 vs. NFD; Figure 1d). Assessment of epididymal adipose tissue, where the organ index (tissue weight/body weight × 100%) reflects systemic lipid storage, showed a significant increase in the HFD group compared to the NFD group (*p* < 0.0001; Figure 1e). This increase was reversed in the L-POST, H-POST, and L-820 groups. Similarly, the weights of the liver and kidney, as major metabolic organs, were significantly increased in HFD mice. The low- and high-dose postbiotics and L820 groups significantly reduced kidney weight (Figure 1f). Liver weights were also significantly reduced in all intervention groups compared with the HFD control group (Figure 1g). These findings collectively suggest that both *L. rhamnosus* IOB820 and its postbiotics exert lipid-lowering and weight-reducing effects through modulation of body weight, glucose homeostasis, and organ indices. Notably, H-POST outperformed the OLYM group in reducing fasting blood glucose concentrations and attenuating kidney and epididymal fat indices.

### 3.2. L. rhamnosus IOB820 and Its Postbiotics Ameliorate Dyslipidemia and Hepatic Metabolic Dysfunction in HFD Mice

**Figure 2:**
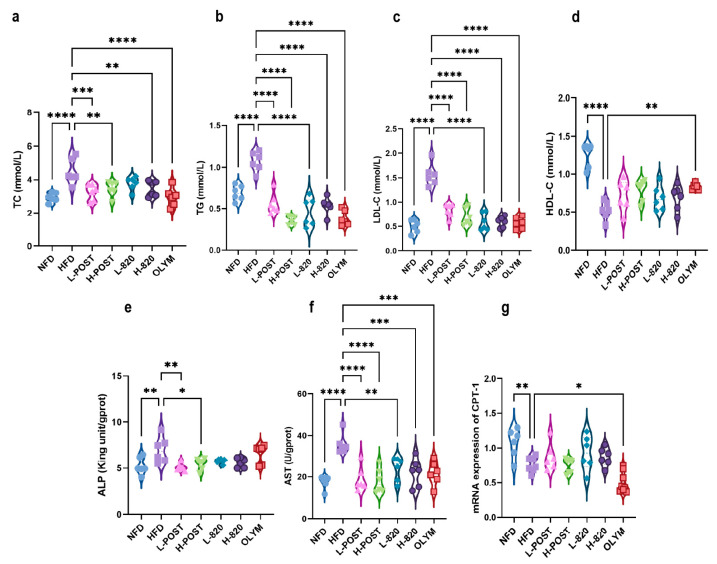
Modulation of Serum Lipid Profiles and Hepatic Metabolic Markers by *L*. *rhamnosus* IOB820 and Postbiotics in HFD Mice. (**a**–**d**) Quantification of serum lipid parameters: TC, TG, LDL-C, and HDL-C; (**e**–**g**) Analysis of hepatic metabolic biomarkers: ALP, AST, and *CPT-1* expression. Statistical significance: * *p* < 0.05, ** *p* < 0.01, *** *p* < 0.001, **** *p* < 0.0001 vs. HFD group, *n* = 6.

Chronic HFD consumption induces obesity and dysregulation of lipid metabolism. To evaluate the therapeutic efficacy of *L. rhamnosus* IOB820 and its postbiotics against HFD-induced dyslipidemia, we quantified four serum lipid parameters: total cholesterol TC, TG, LDL-C, and HDL-C. As shown in Figure 2b,c, all intervention groups significantly reduced the levels of TG and LDL-C induced by the high-fat diet. In Figure 2a, all groups, except for the L820 group, significantly reduced TC levels. When assessing HDL-C, a significant increase was observed only in the OLYM group.

In HFD mice, elevated serum AST and ALP levels are indicative of hepatic injury and impaired bile acid metabolism. *CPT-1*, a pivotal regulator of hepatic β-oxidation [34], showed significantly reduced expression in HFD mice, a deficiency that exacerbates insulin resistance and systemic metabolic disturbances. Figure 2e–g show that HFD mice had significantly higher hepatic AST and ALP levels (*p* < 0.0001 and *p* < 0.01 vs. NFD, respectively) and markedly reduced *CPT-1* expression (*p* < 0.01 vs. NFD). Postbiotic interventions, especially at high doses, effectively attenuated the HFD-induced increases in AST/ALP levels and the suppression of *CPT-1* expression. These findings collectively indicate that HFD consumption disturbs systemic lipid homeostasis and hepatic metabolic function. Crucially, postbiotics formulations—especially high-dose preparations—more effectively inhibited dyslipidemia and hepatic metabolic dysfunction than live bacteria or orlistat, as evidenced by their superior normalization of pathological biomarkers.

### 3.3. L. rhamnosus IOB820 and Its Postbiotics Attenuate Obesity-Associated Inflammation and Tissue Damage in HFD Mice

**Figure 3:**
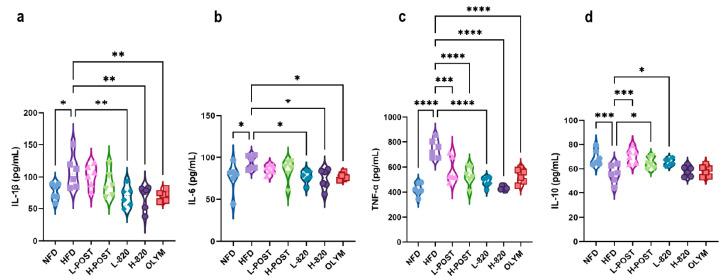
*L. rhamnosus* IOB820 and Postbiotics Modulate Serum Inflammatory Cytokine Profiles in HFD Mice. (**a**–**d**) Quantitative analysis of serum pro- and anti-inflammatory mediators: IL-1β, IL-6, IL-10, and TNF-α. Statistical significance: * *p* < 0.05, ** *p* < 0.01, *** *p* < 0.001, **** *p* < 0.0001 vs. HFD group, *n* = 6.

Obesity induces a chronic low-grade systemic inflammatory state, wherein adipose tissue dysfunction leads to abnormal secretion of adipokines (e.g., leptin and adiponectin) and pro-inflammatory cytokines such as TNF-α, IL-6, and IL-1β. Excessive lipid accumulation exacerbates pro-inflammatory adipokine secretion while downregulating anti-inflammatory mediators such as IL-10, resulting in a pathological imbalance. This imbalance can be ameliorated by therapeutic interventions. As shown in Figure 3a–d, serum concentrations of TNF-α, IL-1β, and IL-6 in HFD mice were significantly elevated, while IL-10 was markedly decreased. Figure 3a demonstrates that both the L-820 and H-820 groups, as well as the OLYM group, significantly attenuated the HFD-induced increase in IL-1β (*p* < 0.01). In Figure 3b, both the low- and high-dose probiotic groups, as well as the OLYM group, significantly reduced the upregulation of IL-6 (*p* < 0.05). Figure 3c shows that all intervention groups significantly suppressed the HFD-induced elevation of TNF-α. Additionally, the L-POST group (*p* < 0.001), the H-POST group (*p* < 0.05), and the L-820 group (*p* < 0.05) were notably effective in restoring IL-10 levels, reversing the downregulation induced by the HFD.

**Figure 4:**
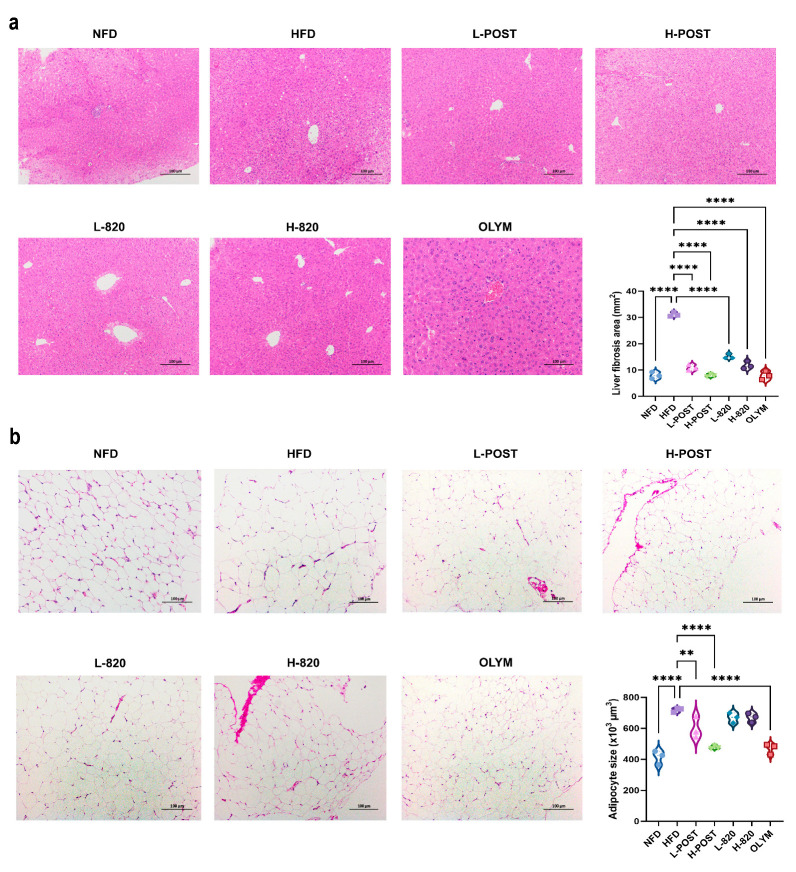
Histopathological Improvements in Hepatic and Adipose Tissues by *L*. *rhamnosus* IOB820 and Postbiotics in HFD Mice. (**a**) Representative HE-stained liver sections (200× magnification); (**b**) Representative HE-stained epididymal adipose sections (200× magnification). ** *p* < 0.01, **** *p* < 0.0001 vs. HFD group.

Elevated inflammatory factors induce tissue damage through multifactorial pathways, including apoptosis, oxidative stress, immune cell activation, and fibrotic remodeling. HE of hepatic and epididymal adipose tissue sections revealed that hepatocytes from the NFD group maintained intact cellular morphology, exhibiting uniform dimensions and radial arrangement around central veins, with no evident lipid vacuoles (Figure 4a). In contrast, HFD mice exhibited marked vacuolar degeneration, perivascular hepatocyte swelling, cytoplasmic rarefaction, disorganized hepatic cord architecture, and extensive microvesicular steatosis when compared to the NFD group (Figure 4a). Therapeutic intervention substantially mitigated these pathological alterations: when compared to the HFD group, treated cohorts exhibited significantly reduced adipocyte cross-sectional areas, decreased lipid vacuolation density, and restored hepatocyte spatial organization with improved size uniformity (Figure 4a). Analysis of epididymal adipose tissue (Figure 4b) further confirmed that the HFD induced prominent adipocyte hypertrophy, while the NFD group maintained uniformly sized, tightly packed adipocytes. Among the treatments, significant reductions in adipocyte size were observed exclusively in the L-POST, H-POST, H-820, and OLYM groups compared to HFD controls, with most treatments also restoring adipocyte size homogeneity. Notably, all intervention groups profoundly alleviated the HFD-induced adipocyte enlargement. These findings collectively demonstrate that *L. rhamnosus* IOB820 and its postbiotics mitigate obesity-associated inflammatory responses in HFD mice, thereby reducing the inflammatory factor-induced damage in hepatocytes and adipose tissue.

### 3.4. L. rhamnosus IOB820 and Its Postbiotics Restore Gut Microbiota Homeostasis in HFD Mice

**Figure 5:**
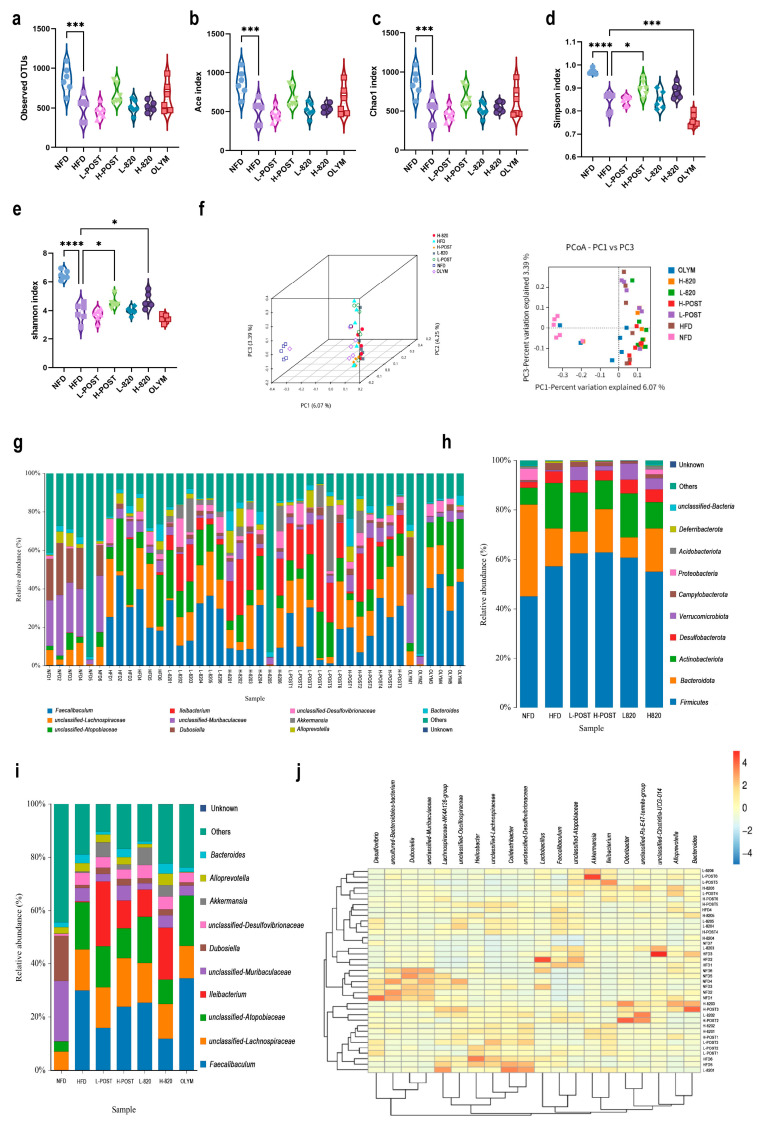
*L. rhamnosus* IOB820 and Postbiotics Modulate Gut Microbiota Composition in HFD Mice. (**a**–**e**) Alpha diversity indices: (**a**) Observed species richness; (**b**) ACE estimator; (**c**) Chao1 richness; (**d**) Shannon diversity index; (**e**) Simpson dominance index; (**f**) PCoA based on Bray-Curtis dissimilarity; (**g**) Taxonomic composition bar plots at genus level; (**h**,**i**) Relative abundance analysis of key bacterial phyla; (**j**) Heatmap visualization of differentially abundant taxa. Statistical significance: * *p* < 0.05, *** *p* < 0.001, **** *p* < 0.0001 vs. HFD group, *n* = 6.

Alpha diversity analysis, including Chao1, Ace, Shannon, and Simpson indices, revealed a significant reduction in gut microbial richness and diversity in HFD mice compared to NFD controls (*p* < 0.001). Intervention groups showed a restoration of alpha diversity metrics (Figure 5a–e). Principal Coordinates Analysis (PCoA) demonstrated clear separation between the NFD and HFD microbiota profiles. All treatment groups occupied intermediate positions along the PCoA gradient, with distinct spatial clustering observed for the positive control (OLYM), probiotics, and postbiotics groups (Figure 5f), indicating differential modulation mechanisms among the interventions.

Taxonomic analysis identified two key alterations in the HFD microbiota: a significant enrichment of *Campylobacterota* (pathobiont) and a depletion of *Verrucomicrobiota* (beneficial phylum). At the genus level, postbiotics interventions specifically and significantly enriched the abundance of *Akkermansia*, a genus within the phylum *Verrucomicrobiota* that is critically associated with metabolic health (Figure 5g–i). Furthermore, the HFD group exhibited an increased abundance of *Atopobiaceae*, which was reversed in the H-POST and H-820 groups. The OLYM group showed no significant change in abundance relative to the HFD group. Notably, *Ileibacterium* levels were elevated in the live bacteria and postbiotic groups, a phenomenon absent in the OLYM group (Figure 5i). These findings collectively demonstrate that *L. rhamnosus* IOB820 and its postbiotics effectively restore gut microbiota homeostasis by suppressing harmful bacterial taxa and enriching beneficial microbial consortia. This microbial remodeling is likely mediated via the gut–liver axis, which may underlie the observed metabolic improvements and amelioration of obesity-associated dysfunctions in HFD mice.

### 3.5. L. rhamnosus IOB820 and Its Postbiotics Modulate SCFA Production in HFD Mice

**Figure 6:**
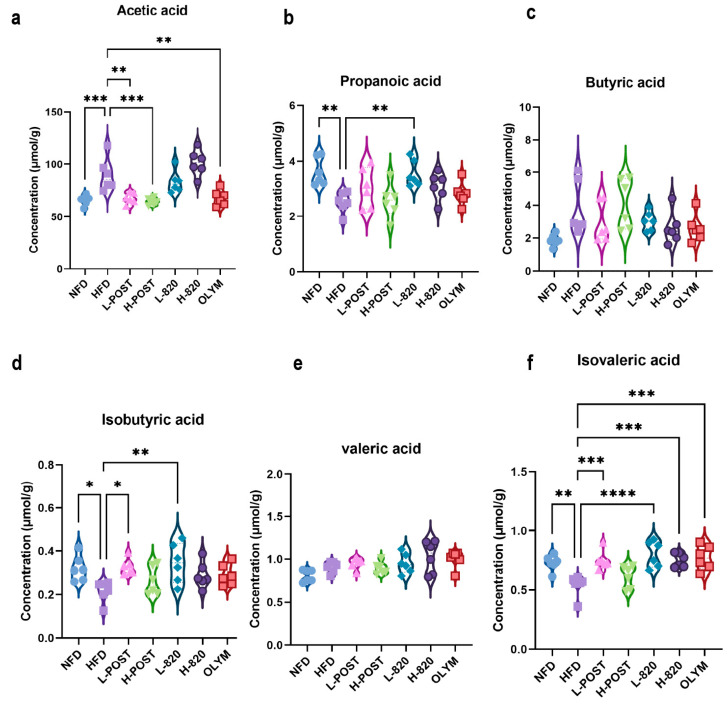
*L. rhamnosus* IOB820 and Postbiotics Reconstitute SCFA Profiles in HFD Mice. (**a**–**f**) Gas chromatography–mass spectrometry (GC-MS) quantification of cecal SCFAs: (**a**) Acetic acid; (**b**) Propionic acid; (**c**) Butyric acid; (**d**) Isobutyric acid; (**e**) Valeric acid; (**f**) Isovaleric acid. Statistical significance: * *p* < 0.05, ** *p* < 0.01, *** *p* < 0.001, **** *p* < 0.0001 vs. HFD group, *n* = 6.

High-fat diet-induced obesity alters gut microbial composition and structure, leading to disruptions in SCFA biosynthesis. SCFAs are key mediators through which gut microbiota regulate hepatic lipid metabolism, inflammation, and fibrotic processes. To investigate the effects of *L. rhamnosus* IOB820 and its postbiotics on SCFA production, we analyzed SCFA levels in the cecal contents of mice. Initial observations revealed trends in how the high-fat diet and its interventions influenced the SCFA profile (Figure 6). Compared to the NFD group, acetate levels were significantly elevated in the HFD group (Figure 6a). However, propionate, isobutyrate, and isovaleric acid levels were significantly decreased, with values in the HFD group markedly lower than in the NFD group, exhibiting minimal overlap in error bars. Notably, the L-820 group showed SCFA levels that were either restored to NFD-like values or even exceeded those in the NFD group, with minimal overlap with the HFD group (Figure 6b,d,f). These results suggest that the interventions may have a meaningful impact on restoring the levels of these three SCFAs. The changes in butyrate and valeric acid levels, however, were more complex (Figure 6c,e), indicating that there were no fundamental differences in butyrate and valeric acid production between the groups. Nevertheless, both the live bacteria and postbiotic interventions, particularly the postbiotic treatment, showed trends toward restoring multiple SCFAs (such as acetate, isobutyrate, and isovalerate) to NFD levels. Propionate and isobutyrate were also detected in the low-dose live bacteria group, which showed a trend toward NFD levels. Furthermore, except for the H-POST group, all other treatment groups exhibited significant recovery in isovaleric acid levels following intervention. Although these findings are preliminary, they align with the microbial shifts observed in 16S rRNA sequencing data. For instance, the recovery of propionate, isobutyrate, and isovaleric acid in the L-820 group may be linked to the increased relative abundance of acidogenesis-associated bacterial groups, such as Verrucomicrobiota, in this group. It is well known that propionic acid and isobutyric acid play important roles in regulating lipid metabolism and immune homeostasis. Therefore, these trends provide mechanistic insights that warrant further investigation to confirm the role of *L. rhamnosus* IOB820 and its postbiotics in improving metabolic health via the “microbiota-metabolites” axis.

### 3.6. Correlation Analysis of Lipid Metabolism, Gut Microbiota and SCFAs

**Figure 7:**
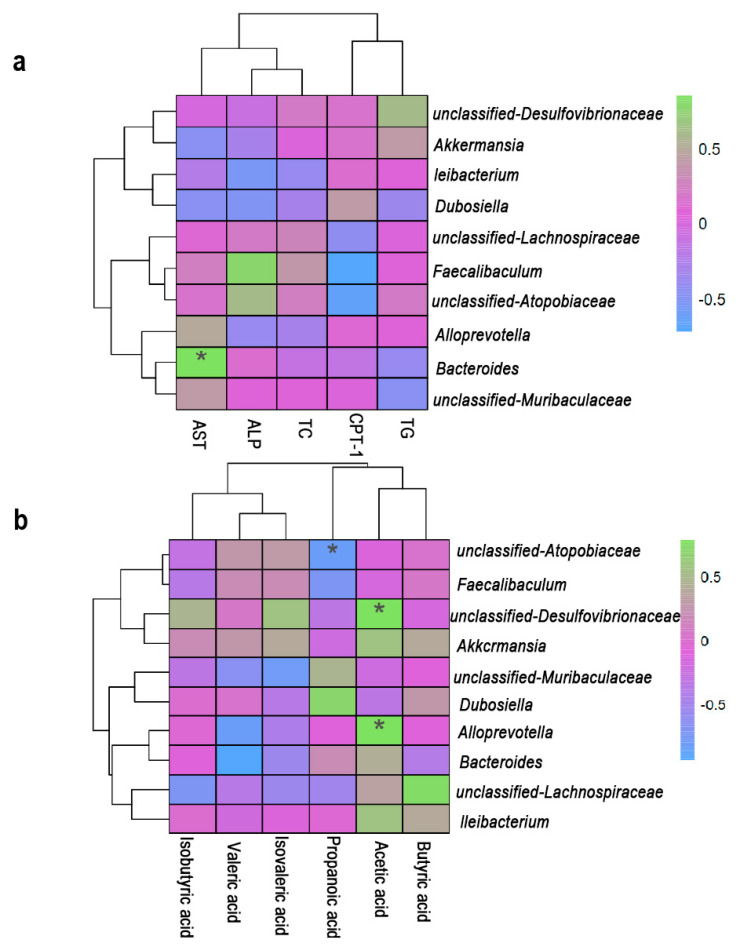
Spearman correlation between lipid metabolism (AST, ALP, TC, TG, *CPT-1*), SCFAs and microbial genera. (**a**) Correlations between lipid metabolism and microbial genera. (**b**) Correlations between SCFAs and microbial genera. Figure 7 was completed using the Wekemo Bioincloud (https:// www.bioincloud.tech accessed on 17 September 2025). * *p* < 0.05.

The findings of this study demonstrate that *L*. *rhamnosus* IOB820 and its postbiotics regulate lipid metabolism-related indicators by modulating gut microbiota composition and mediating SCFA levels, thereby exerting a significant anti-obesity effect in HFD-induced obese mice. To further explore the interrelationships between gut microbiota, lipid metabolism-related indicators, and SCFAs, we conducted a correlation analysis. As shown in Figure 7a, *Bacteroides* was significantly positively correlated with AST, while Figure 7b revealed a significant negative correlation between *Bacteroides* and valeric acid, suggesting that these factors may interact to improve lipid metabolism in HFD mice. This finding is consistent with the study by Xinhao Duan et al. [35]. Additionally, Figure 7b illustrates a significant negative correlation between *Atopobiaceae* and propionic acid, suggesting that propionic acid may help reduce the abundance of *Atopobiaceae*. This observation aligns with the research of Zichen Ning et al. [36]. Furthermore, Figure 7b shows that acetic acid was significantly positively correlated with *Alloprevotella* and unclassified *Desulfovibrionaceae*. Studies by Botao Wang and Wang Xue et al. also reported that the abundance of unclassified *Desulfovibrionaceae* and *Alloprevotella* in HFD mice could be reversed by increasing SCFA levels [37,38].

**Figure 8:**
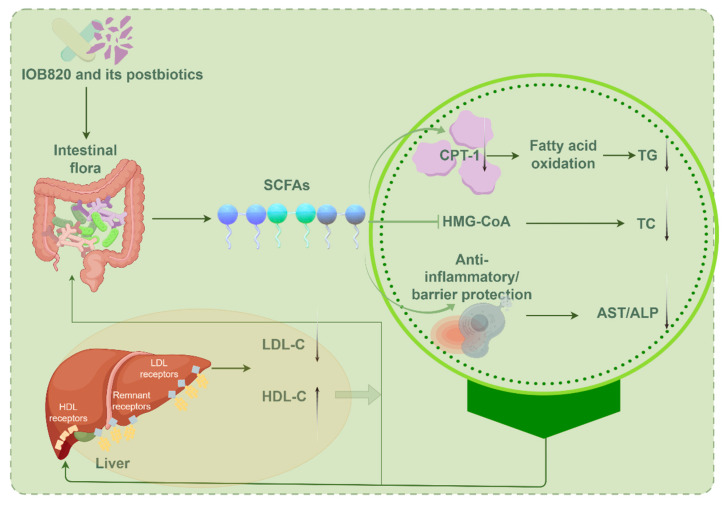
Correlation analysis between intestinal flora and lipid metabolism. Produced through https://www.figdraw.com accessed on 7 August 2025. Thanks are given to Figdraw.

The above studies highlight a strong correlation between gut microbiota, SCFAs, and lipid metabolism. Based on the observed changes in microbial composition and SCFA profiles, alongside insights from the literature, we propose a hypothetical mechanistic model (Figure 8). However, the causal relationships within this model require further experimental validation. Specifically, the restoration of propionate and isovalerate levels may upregulate Cpt1a mRNA expression, promoting fatty acid β-oxidation and reducing TG levels (Figure 2b and Figure 6). Although no significant changes in butyrate levels were observed, its potential role as a histone deacetylase (HDAC) inhibitor could contribute to metabolic regulation, as noted by Hou Gai-feng et al. [39]. Moreover, the reduction in TC production (Figure 2a) may result from the inhibition of HMG-CoA reductase, the rate-limiting enzyme in cholesterol synthesis, as confirmed by Jingnan Chen et al. [40].

Hepatic inflammation is a key contributor to elevated AST and ALP levels. Immune cells infiltrate the liver, releasing proinflammatory cytokines that damage liver cell membranes, causing the leakage of AST and ALT into circulation. SCFAs may mitigate this by enhancing intestinal barrier function or inhibiting inflammatory cytokines like TNF-α and IL-6, thereby reducing serum AST and ALP levels. Additionally, SCFAs can influence liver LDL and HDL receptors, regulating the expression of LDL-C and HDL-C, which in turn impacts the gut microbial composition. This dynamic feedback loop, involving gut microbiota, SCFAs, and host metabolic indicators, provides a novel perspective and therapeutic target for metabolic diseases. It offers new strategies for addressing obesity, diabetes, nonalcoholic fatty liver disease (NAFLD), and cardiovascular diseases (CVD), while also deepening our understanding of the gut–liver axis in inflammatory bowel disease (IBD). These findings not only advance our knowledge of the “microbiome-metabolism” axis but also lay the foundation for microbiome-based precision treatments.

## 4. Discussion

Obesity has become a major global health challenge, demanding sustainable, accessible, and effective treatments to mitigate this public health crisis. Probiotics, defined as symbiotic microorganisms that colonize the human intestine, are widely used to prevent and treat various diseases by excluding pathogens, enhancing intestinal barrier function, and regulating immune responses [41,42]. Growing evidence from both animal and human studies confirms that probiotics can ameliorate metabolic disorders, inflammation, and excessive weight gain in obese individuals [43]. Although obesity’s etiology involves multifactorial mechanisms, research strongly links gut microbiota dysbiosis to its pathogenesis and metabolic complications [44]. Modulating the intestinal microbiome to restore metabolic balance has therefore emerged as a potential strategy for lipid-lowering and weight-loss interventions.

We measured four blood lipids and liver metabolism-related indicators. The results showed that the levels of TC, TG, LDL-C, ALP, and AST in mice following intervention were significantly lower compared to the HFD group, while HDL-C and *CPT-1* levels were higher. This is consistent with Ahmed et al. [45]. Notably, changes in *CPT-1* mRNA expression may not directly reflect changes in protein abundance or enzymatic activity, and future studies incorporating Western blotting and enzyme assays are needed for functional validation. Additionally, ALP test results indicated that *L. rhamnosus* IOB820 postbiotics had a more pronounced therapeutic effect on HFD mice compared to the positive drug OLYM. However, in Abbas MA et al.’s study on brown algae extract, the reduction of ALP was most notable with the use of positive drugs [46].

In line with these metabolic improvements, *L. rhamnosus* IOB820 and its postbiotics significantly attenuated chronic low-grade inflammation characteristic of obesity. The HFD-induced increase in pro-inflammatory cytokines (IL-1β, IL-6, TNF-α) was suppressed by our interventions, which also enhanced the anti-inflammatory mediator IL-10. Histopathological analyses confirmed a marked reduction in hepatic steatosis and adipocyte hypertrophy in treated groups. Similarly, Zhang et al. demonstrated that *Limosilactobacillus reuteri* J1 prevents weight gain, reduces fat mass, and improves glucose homeostasis and insulin sensitivity in HFD mice [47]. We then sought to examine whether these metabolic and anti-inflammatory benefits were linked to gut microbiota restructuring. Our data confirmed that *L. rhamnosus* IOB820 and its postbiotics restored gut microbiota homeostasis disrupted by HFD. This was evidenced by increased alpha diversity, a reduction in pathobionts such as *Campylobacterota*, and a significant enrichment of beneficial genera, particularly *Akkermansia*. This microbial remodeling was accompanied by a reconstitution of the gut metabolome, notably restoring SCFA production, including propionate, isobutyrate, and isovalerate. These microbially derived metabolites provide a plausible mechanism linking gut microbiota changes to improved metabolism. Specifically, *L. rhamnosus* IOB820 appears to regulate lipid metabolism by modulating the production of propionate, isobutyrate, and isovaleric acid. SCFAs activate signaling pathways through GPR43 and GPR41, with propionate notably influencing lipid metabolism [48,49]. Furthermore, the findings for isobutyrate and isovaleric acid are consistent with the work of Liang He [50] and Yishu Chen [51].

Haiyu Zhang et al. demonstrated that traditional Chinese medicine could improve metabolic diseases by modulating the intestinal microbiota, stimulating SCFA-producing bacteria to enhance host metabolism and inflammation [52]. Haoran Chen et al. also showed that *Lactobacillus plantarum* HF02 improves lipid accumulation and gut microbiota imbalance in HFD-induced obese mice, increasing fecal SCFA levels [53]. These studies reinforce the link between intestinal microbiota, SCFAs, and lipid metabolism, suggesting these mechanisms as potential therapeutic targets for metabolic diseases. Nehmi et al. also found that nutritional supplements containing yeast β-glucan, prebiotics, minerals, and silymarin (silybin) improved obesity-related metabolic parameters in humans [54]. These findings provide valuable theoretical support for advancing such supplements into clinical trials. However, further work is necessary to ensure that probiotic therapies meet the safety, purity, and potency standards required for medical use.

A key finding of this study is that *L. rhamnosus* IOB820 postbiotics exhibited comparable, or even superior, effects to live bacterial preparations in improving various obesity-related indicators. This highlights the potential of postbiotics as a new microbial therapy. Postbiotics offer several advantages over traditional probiotics, including excellent safety, stability, clear mechanisms of action, and ease of processing and application [55,56]. The significant effects observed in the high-dose postbiotic group (H-POST) further support the potential of *L. rhamnosus* IOB820 postbiotics as a safer, more stable, efficient, and convenient anti-obesity functional preparation. This opens new avenues for dietary interventions and personalized nutrition strategies for obesity.

While this study provides compelling evidence of the efficacy and mechanisms of *L. rhamnosus* IOB820 and its postbiotics in ameliorating obesity and metabolic disorders, several limitations remain. First, we used 16S rRNA gene sequencing to analyze changes in gut microbiota composition. While this method is effective for composition analysis, it does not provide functional gene-level information. Future studies should employ metagenomic techniques to elucidate specific microbial metabolic pathways modulated by these interventions. Second, the daily energy intake of mice was not monitored, so potential differences in food intake between groups cannot be ruled out as a factor influencing body weight and metabolic outcomes. However, the observed dose-dependent effects and specific remodeling of the gut microbiota and SCFAs suggest that the effects are not merely due to nonspecific appetite suppression. Finally, the physiological differences between mice and humans in gut microbiota composition, physiology, and dietary environment require further clinical validation of these findings, particularly regarding changes in specific bacterial genera.

## 5. Conclusions

In summary, this study demonstrates that *L. rhamnosus* IOB820 and its postbiotics significantly mitigate HFD-induced obesity and metabolic comorbidities in mice. The interventions improved weight gain, lipid homeostasis, inflammation, and gut microbiota balance, with postbiotics showing comparable or superior efficacy to live probiotics and orlistat. These results highlight the gut microbiota–metabolites axis as a promising target for obesity treatment, laying the groundwork for future studies and clinical trials. Live probiotics could be developed in forms like freeze-dried capsules, powders, and functional food additives.

## Figures and Tables

**Table 1:** Sequences of RT-qPCR-specific primers.

Target Gene	Forward Primer	Reveres Primer
*GAPDH*	ATGGTGAAGGTCGGTGTGAACGG	TGGAACATGTAGACCATGTAGTGAGG
*CPT-1*	CAAGAACAGCAACGAGTACCG	GTCACTGGTCAACTCCAGCAC

## Data Availability

The original contributions presented in this study are included in the article. Further inquiries can be directed to the corresponding authors.

## Abbreviations

The following abbreviations are used in this manuscript:

HFD	High-fat diet
SCFA	Short-chain fatty acid
NFD	Normal diet
L-POST	Low-dose postbiotics
H-POST	High-dose postbiotics
OLYM	Orlistat-treated positive control
HDACs	Histone deacetylases
SPF	Specific pathogen-free
LDL-C	Low-density lipoprotein cholesterol
HDL-C	High-density lipoprotein cholesterol
TC	Total cholesterol
TG	Triglycerides
TNF-α	Tumor necrosis factor-α
IL-10	Interleukin-10
IL-6	Interleukin-6
IL-1β	Interleukin-1β
AST	Aspartate aminotransferase
ALP	Alkaline phosphatase
GAPDH	Glyceraldehyde-3-phosphate dehydrogenase
CPT-1	Carnitine acyl transferase I
PCoA	Principal Coordinates Analysis
NAFLD	Nonalcoholic fatty liver disease
CVD	Cardiovascular disease
IBD	Inflammatory bowel disease
